# An Interesting Case of Cholangitis

**DOI:** 10.7759/cureus.60537

**Published:** 2024-05-18

**Authors:** Sontosh Reddy, Archith Boloor, Nikhil Kenny Thomas

**Affiliations:** 1 Internal Medicine, Kasturba Medical College, Mangalore, Mangalore, IND; 2 Gastroenterology, PSG Institute of Medical Sciences & Research, Coimbatore, IND

**Keywords:** severe ards, severe sepsis, acute cholangitis, ascariasis, biliary diseases

## Abstract

Ascariasis is one of the most common parasitic infections in the world. It is mostly asymptomatic; however, rarely when the worms migrate to the biliary tract, they can cause biliary ascariasis. It typically presents with pain abdomen, jaundice, and fever. This case report is about a patient who presented with fever, icterus, breathlessness, loose stools, and altered sensorium but had no abdominal pain. The patient was diagnosed with biliary ascariasis using ultrasound and endoscopic retrograde cholangiopancreatography (ERCP). The patient was treated with endoscopic sphincterotomy and albendazole. The patient remained stable after 10 days. The absence of abdominal pain highlights the variability of the presentation of biliary ascariasis.

## Introduction

Approximately 807 million to 1.2 billion people worldwide are infected with Ascaris lumbricoides [1]. It is commonly seen in developing countries in regions of low socio-economic status and is transmitted by ingesting infective eggs. The parasite usually resides in the small intestine and is often asymptomatic. The most common complaints associated with Ascariasis are abdominal pain, bloating, nausea, vomiting, anorexia, and intermittent diarrhea. However, rarely, it enters the biliary lumen to cause biliary ascariasis. In a patient with biliary Ascariasis, the main symptoms are biliary colic, fever, and icterus; however, in rare cases, they may present in an atypical manner, and only a few such cases have been reported. Some of the complications caused by Ascariasis are biliary colic (the most common presentation), cholangitis, cholecystitis, and rarely pancreatitis or hepatic abscess [2].

## Case presentation

**Figure 1:**
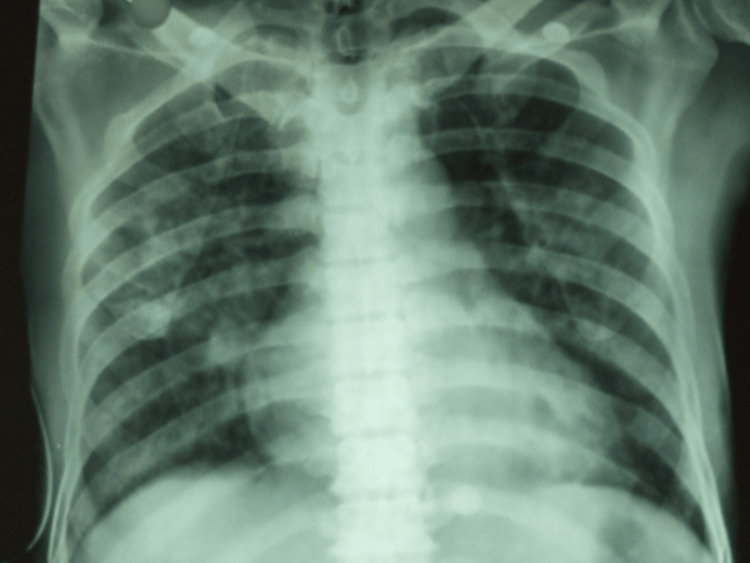
Chest X-ray with bilateral ground glass opacities

A 38-year-old male, from a town in Karnataka, India, presented with complaints of high-grade fever for one week, yellowish discoloration of eyes and urine for five days, altered sensorium for four days, loose stools for four days, and breathlessness for two days. He had no history of vomiting, abdominal pain, bleeding tendencies, cough with expectoration, headache, or neurological deficits and no relevant past history, except for consuming alcohol twice a week for 10 years. On examination, he was disoriented with icterus present, but no signs of liver failure, pallor, cyanosis, clubbing, lymphadenopathy, or edema were observed. His pulse was 106/min, BP was 100/60 mm Hg, SpO2 was 85% at room air, respiratory rate was 36/min, and the temperature was 101°F. Tender hepatomegaly was noted, and the liver could be felt 2 cm below the coastal margin. Coarse crepitations were heard bilaterally in the infraxillary and infrascapular regions. Other systems were unremarkable. The differentials considered were cholangitis, leptospirosis, complicated malaria, alcoholic hepatitis with hepatic encephalopathy, and aspiration pneumonia. Malaria parasites and leptospirosis were ruled out. Laboratory examinations demonstrated a total white blood cell (WBC) count of 16,200/mm^3^, platelets of 56,000/mm^3^, left shift up to band forms, and neutrophilic leukocytosis in the peripheral smear, IgM for leptospirosis (negative), blood urea of 170 mg/dL, serum creatinine of 2.5 mg/dL, amylase of 217 U/L, total bilirubin of 12.4 mg/dL, direct bilirubin of 12.1 mg/dL, albumin of 2.1 g/dL, serum aspartate aminotransferase (AST) of 1146 U/L, alanine aminotransferase (ALT) of 401 U/L, alkaline phosphatase (ALP) of 784 U/L, INR of 2.1, and ammonia of 230 µ/dL. The patient's X-ray was suggestive of acute respiratory distress syndrome (ARDS), indicating sepsis (Figure 1).

**Figure 2:**
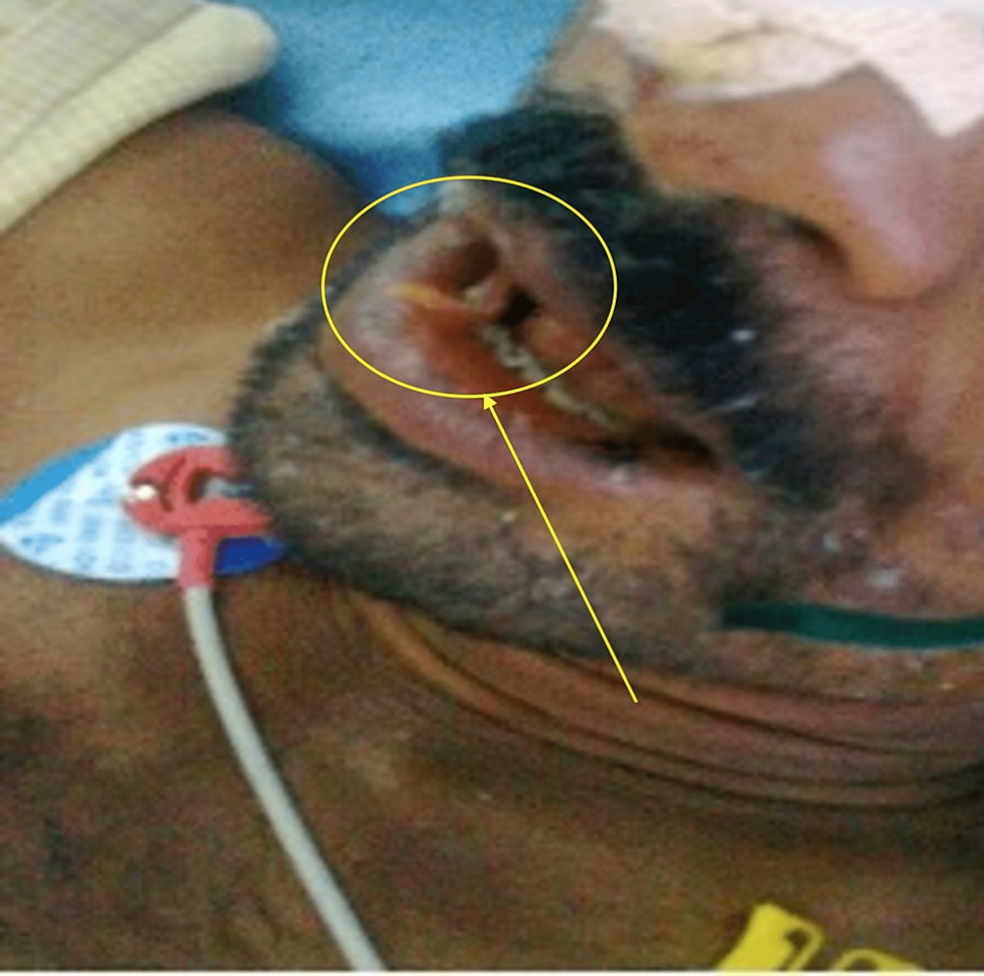
Ascaris lumbricoides worms creeping out of the patient's mouth

**Figure 3:**
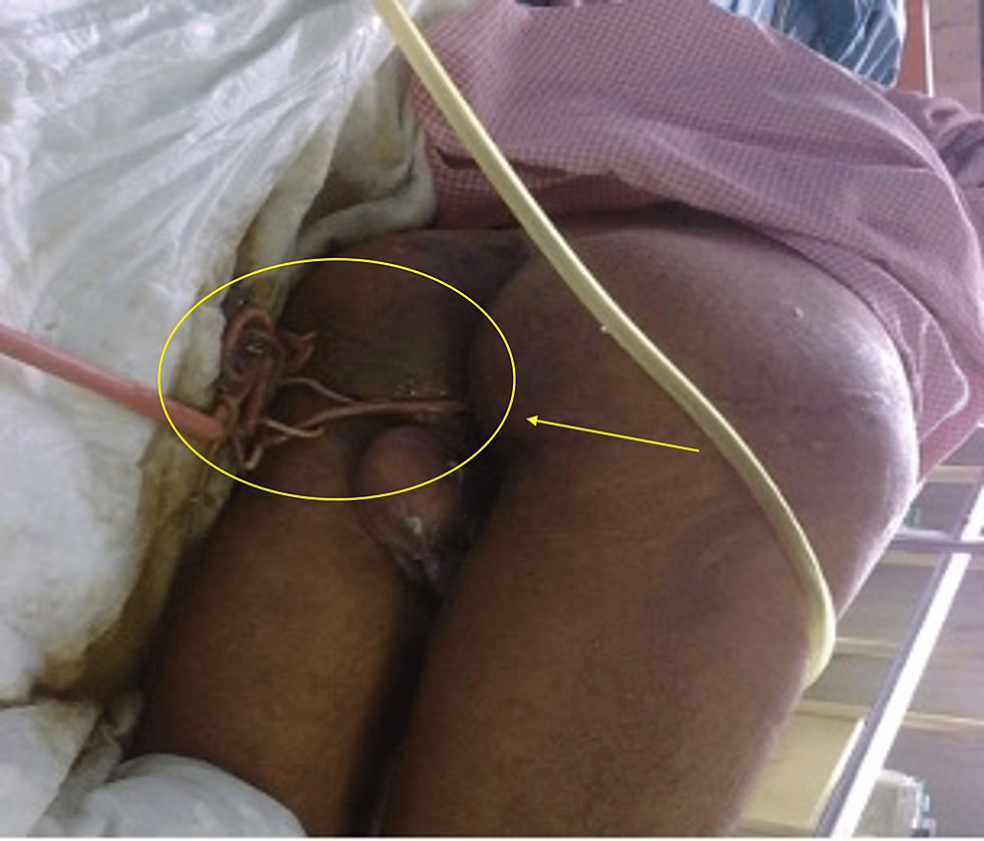
Ascaris lumbricoides coming out of the patient's anal opening

An ultrasound of the abdomen showed intrahepatic biliary radicle dilatation and hyperechoic tubular structures in the common bile duct and gallbladder. The patient was being treated with IV fluids and an injection of ceftriaxone, and anti-encephalopathy measures were given. During the treatment, worms started coming out of the patient’s mouth (Figure 2) and anal opening (Figure 3).

An endoscopic retrograde cholangiopancreatography (ERCP) was done, which showed multiple worms in the esophagus and stomach, but the common bile duct was empty and normal in size. A small biliary sphincterotomy was done. He was then started on T. albendazole 400 mg once daily (OD), for three days, and 45-50 dead worms were passed after bowel washes and 10-15 worms orally over the next five days. After 10 days, his investigations came back to normal to total bilirubin of 2.41 mg/dL, direct bilirubin of 1.31 mg/dL, creatinine of 0.7 mg/dL, ALP of 191 U/L, and AST of 83 U/L, and he was discharged. There has been no recurrence so far.

## Discussion

This case highlights an atypical presentation of biliary ascariasis. There has been no case report where biliary ascariasis has presented without abdominal pain and with altered sensorium and dyspnea. Ascariasis is the most common helminthic infection in the world [3]. The prevalence of ascariasis in the adult population in an endemic area was 30% according to a recent random survey [4]. In a study, they found that the overall prevalence of biliary ascariasis was 0.45% [5]. In humans, the life cycle of this parasite begins after the ingestion of its egg, following which larvae hatch and invade the small bowel mucosa, enter the systemic circulation, go to the lungs, ascend the tracheobronchial tree, and are then swallowed to enter back into the intestine where they mature into adult worms [6]. Intestinal infections are common and usually asymptomatic. When the worm load is heavy during the process of larval migration, the larvae may enter the biliary tract, leading to a condition termed biliary ascariasis. Biliary ascariasis can have varied presentations from biliary colic being the most common to obstructive jaundice, cholangitis, cholecystitis, and rarely pancreatitis or hepatic abscess [7]. After reviewing reports from various sources, we found that pain in the abdomen associated with jaundice and fever were the presenting complaints of this condition. The diagnostic modality of choice is usually an ultrasound abdomen with 86% sensitivity in cases of a single worm and 100% in cases of multiple worms in CBD [8]. This condition can be managed conservatively with albendazole and IV antibiotics, but in the presence of a worm inside the gall bladder, an endoscopic sphincterotomy yields good results. If any dead worms are present, they also should be removed. The diagnosis of biliary ascariasis in the patient was established through the use of ultrasound, and the presence of worms being expelled from the patient's mouth and anus. This diagnosis was later confirmed by ERCP, which identified the presence of multiple Ascaris worms in the esophagus and stomach. A small biliary sphincterotomy was performed to treat the condition. We finally concluded the diagnosis to be biliary ascariasis with septic/hepatic encephalopathy with ARDS. The patient was started on T. albendazole 400 mg OD for three days. Additionally, 45-50 dead worms passed after bowel washes, and 10-15 worms passed orally over the next five days. After 10 days, the patient was stable, and his laboratory values returned to normal.

## Conclusions

Typical clinical presentation for biliary ascariasis is icterus, pain abdomen, and fever. Biliary ascariasis can present atypically as in our case without pain abdomen, breathlessness, and altered sensorium. Biliary ascariasis should be considered as a differential, in any case presenting with jaundice and fever even if abdominal pain is absent. If left unrecognized and untreated early, it can lead to complications and multiple end-organ damages, including sepsis.

## References

[1] (2022). About ascariasis. https://www.cdc.gov/parasites/ascariasis/index.html.

[2] Pilankar KS, Amarapurkar AD, Joshi RM, Shetty TS, Khithani AS, Chemburkar VV (2003). Hepatolithiasis with biliary ascariasis--a case report. BMC Gastroenterol.

[3] Bethony J, Brooker S, Albonico M, Geiger SM, Loukas A, Diemert D, Hotez PJ (2006). Soil-transmitted helminth infections: ascariasis, trichuriasis, and hookworm. Lancet.

[4] Khuroo MS, Mahajan R, Zargar SA, Javid G, Sapru S (1989). Prevalence of biliary tract disease in India: a sonographic study in adult population in Kashmir. Gut.

[5] Akhter N, Islam SM, Mahmood S, Hossain GA, Chakraborty RK (2006). Prevalence of biliary ascariasis and its relation to biliary lithiasis. J Med Ultrason (2001).

[6] Weller PF, Nutman TB (2018). Intestinal nematodes. Harisson's Principles of Internal Medicine, 20e.

[7] Khuroo MS, Zargar SA, Mahajan R (1990). Hepatobiliary and pancreatic ascariasis in India. Lancet.

[8] Misra SP, Dwivedi M (2000). Clinical features and management of biliary ascariasis in a non-endemic area. Postgrad Med J.

